# The Effects of Decreasing Dietary Crude Protein on the Growth Performance, Feed Efficiency and Meat Quality of Finishing Charolais Bulls

**DOI:** 10.3390/ani9110906

**Published:** 2019-11-01

**Authors:** Martina Cortese, Severino Segato, Igino Andrighetto, Nicola Ughelini, Maria Chinello, Eliana Schiavon, Giorgio Marchesini

**Affiliations:** 1 Departement of Animal Medicine, Production and Health, University of Padova, 35020 Legnaro (PD), Italy; martina.cortese.1@studenti.unipd.it (M.C.); severino.segato@unipd.it (S.S.); igino.andrighetto@unipd.it (I.A.); nicola.ughelini@gmail.com (N.U.); maria.chinello@unipd.it (M.C.); 2 Istituto Zooprofilattico Sperimentale delle Venezie, 35020 Legnaro (PD), Italy; eschiavon@izsvenezie.it

**Keywords:** finishing beef cattle, dietary protein, digestibility, meat quality, economic assessment

## Abstract

**Simple Summary:**

Livestock production emerges as one of the main contributors of ammonia emissions; in fact, as the literature reports, the excess of nitrogen fed in form of feed protein is excreted in manure and converted into ammonia. Defining beef cattle protein requirements, specifically for each breed and farming system, is fundamental to ensuring an adequate supply of protein, while avoiding N losses due to an over-estimation of needs. In this study, we compared two different levels of protein in beef cattle diets to better understand the exact amount that would meet the animals’ requirements, while avoiding waste. Results showed that, on one hand, the decrease of protein in the diet could actually compromise animals’ daily weight gains, but on the other hand, it did not reduce the income of the farmer, because the diet was cheaper, and improved the efficiency in the use of the digestible protein for growth.

**Abstract:**

This study aimed to evaluate the effect of decreasing dietary crude protein (CP) on the performance of finishing Charolais bulls in the Italian rearing system. Animals were fed two diets, differing only in the CP level (low protein (LP), 13.5% CP versus control (CON), 15.0% CP). Dry matter (DM) intake (DMI) and animals’ weights were recorded to obtain average daily gain (ADG) and feed conversion rate (FCR). Feed and fecal samples were collected to evaluate digestibility of diet components. Daily cost of the ration (DRC), feed cost per kg of daily weight gain (CDG) and daily gross margin (DGM) were calculated to analyze the possible benefits of decreasing the protein level. Meat quality analyses were also conducted. Higher DMI (10.6 versus 10 kg/d; *p* < 0.05) and ADG (1.47 versus 1.36 kg/d; *p* < 0.05) were observed for CON. No differences in FCR or digestibility were found. Even if the DRC was lower (*p* < 0.05) for the LP diet (2.26 versus 1.97 €; CON versus LP), no difference was reported for CDG and DGM. Meat lightness and redness were significantly lower and higher in the LP, respectively. To conclude, the CP requirement in these rearing conditions appeared to be higher than 13.5%.

## 1. Introduction

The livestock sector, in particular beef cattle production, is considered one of the major contributors to environmental impact, and especially, to greenhouse gas (GHG) emissions [1]. The beef supply chain is responsible for the production of large quantities of manure, which is one of the main sources of ammonia. Ammonia (NH_3_) emissions from cattle are related to manure storage, and even more to its subsequent distribution into the land, and are associated with different ecosystem and human health concerns [2]; they are also responsible for soil acidification, as pointed out by Brunet, 1998 [3]. The release of ammonia from manure depends on its content of nitrogen, which in turn is related to dietary crude protein (CP) concentration and digestibility [4,5,6,7]. It has been pointed out that the reduction in the content of nitrogen in manure linearly decreases the emission of ammonia [8]. Previous studies have shown that increasing CP from 11.5% to 13.0% increases emissions of ammonia from 60% to 200% [9]. According to the NRC (2000) [10], CP requirements decrease as animals mature; it may be possible to decrease N losses and nutrient excretion by decreasing CP concentrations without negatively affecting growth rate [11].

Management characteristics and rearing systems of beef cattle production are fairly different among breeds, countries and regions. In particular, in Italy about two thirds of the young bulls are reared by specialized fatteners located mainly in the northern plains of the country (Veneto and Po’ Valley) [12,13]. Charolais bulls are the most dominant category of animals imported in Veneto region and the rearing system is characterized by being divided into four main feeding phases: backgrounding, transition, growing and finishing [14], which are characterized by different CP and energy levels of the ration, in order to meet animals’ requirements [15]. Although the CP requirements to maximize average daily gain (ADG) in growing beef cattle, according to NRC (2000) [10], are 13.8% on dry matter (DM) basis, especially for Angus breed, in Italy the range of CP used on growing and finishing cattle, varies a lot, from 11.0% to 14.7% DM, leading to very different levels of performance and feed efficiency [16,17]. Protein requirements during different growing phases are strongly influenced by many factors, such as feed digestibility, heat increment, tissue metabolism, protein turnover, feeding behavior and activity [18], which makes it hard to understand well, the real CP requirements of beef cattle. Despite the literature present on the general CP requirements of beef growing cattle, to maximize animal performance and the feed efficiency there is the need for studies focused on the requirements of each breed raised in specific conditions.

Moreover, protein is the most expensive component of beef cattle’s ration and if the rate of protein degradation is higher than the rate of carbohydrate fermentation, high quantities of nitrogen are lost as ammonia [19]. Thus, feeding an excess of protein contributes to increased production costs and results in adverse environmental impacts and in the onset of metabolic diseases [20]. Considering the high cost of feeding in beef cattle production and the low profitability of this rearing system, it is critical to improve management and feed efficiency to reduce environmental impacts, minimize nutrient losses and protect animal health. It is necessary to take into consideration that decreasing the CP level can potentially decrease the atmospheric losses of NH_3_, but at the same time it can lead to adverse effects on feed intake and growth performance [21]. In addition, there are many biological processes involved in the expression of meat quality, which highlight the connection between production factors (age, ration composition, breed and feeding system), as well as meat tenderness, marbling and juiciness [22].

All considered, the aim of this study was to assess the effect of reducing the CP level of the ration on growth performance, feed digestibility, feed efficiency, meat quality and the producing costs of Charolais bulls raised during the growing and finishing phases of a typical Italian beef cattle rearing system.

## 2. Materials and Methods

### 2.1. Experimental Groups and Feeding

Experimental procedures were approved by the Organismo Preposto al Benessere Animale—Animal Welfare Group at ISS (OPBA) committee of Padova University (approval number 459116).

A group of Charolais bulls (*n* = 107 animals) was imported from France and raised in a beef cattle farm located in Rovigo province, in North-Eastern Italy, from October 2017 until May 2018, for 207 days, before being sent to the slaughterhouse. Bulls were selected and bought at a cattle collection center in France, based on their weight, age and physical conformation. Upon arrival, animals were on average 300 ± 53 days old and weighed 407 ± 23 kg. Before starting the trial, the young bulls spent the backgrounding and transition phases (in total 45 days) in a loose housing facility with straw bedding and were fed the same diet (Table 1), that was gradually changed throughout the two phases from initial contents of neutral detergent fiber (NDF), crude protein and starch of 38.2%, 14.1% and 26.1% DM, respectively, to 35.2%, 14.0% and 29.8% DM, respectively. The young bulls were reared in 11 contiguous pens, grouped by initial body weight (BW) and age, to guarantee homogeneity within pens, and then, after the transition phase, were moved into a roofed, loose housing facility with concrete-slatted floor for the growing (77 d) and finishing (85 d) phases. To avoid increasing the fights between animals, the groups corresponding to the individual pens were maintained, and these groups were distributed so as to form two macro-groups, balanced for live weight (484 ± 34 kg). The two groups were fed two diets formulated to differ only for CP content: the control diet (*n* = 58; six pens) with 15.0% CP (CON) and the low protein diet (*n* = 49; five pens) with 13.5% CP (low protein, LP). Diets were offered as total mixed rations (TMR) which included corn silage, pressed sugar beet pulp, wheat bran, corn meal, soybean meal, straw, vitamins and minerals, as reported in Table 1. To meet the animals’ requirements, rations were gradually adjusted, moving from a growing phase (P1) to a finishing phase (P2), as shown in Table 1. Young Bulls were fed ad libitum one of two diets, and the offered amount was calculated, aiming for 5% refusals. TMR was distributed once a day, at 10:00 in the morning and there was free access to fresh water, supplied through two pressure bowls per pen.

### 2.2. Animal Monitoring, Sample Collection and Analyses

The trial lasted 162 days and all animals in each pen were individually weighed at the beginning of the trial, between P1 and P2, and at the end of the trial, before TMR distribution, to calculate the individual ADG. Upon arrival, bulls were vaccinated and treated for external and internal parasites, and since the beginning of the trial, they had been checked by trained farm personnel and by a veterinarian twice daily, to verify their health status. Dry matter intake (DMI) of each pen was reckoned by difference between the offered TMR and 24 h leftovers [13], and the feed conversion ratio (FCR) of each pen was calculated dividing the average ADG by DMI. Moreover, to calculate fecal CP output and total tract digestibility of DM (DMD), CP (CPD), NDF (NDFD) and starch (starch_D), fresh fecal samples were gathered from two young bulls per pen, at two-week intervals. Samples of TMR and leftovers of both diets were collected weekly and subjected to chemical analysis. For both TMR and feces, dry matter (DM), ash, CP, NDF, ADL and starch were determined following the procedures reported in literature [23,24,25,26], respectively. Total tract digestibility was calculated using lignin as internal marker. The mathematical equations reported by McDonalds [27] and Schäfers [28] were used to calculate DMD, CPD, NDFD and starch_D.

$$\mathrm{DMD}=\frac{marker\;in\;faeces\;\left(g/kgDM\right)\;-\;marker\;in\;food\;\left(g/kgDM\right)}{marker\;in\;faeces\;\left(g/kgDM\right)},$$

$$\mathrm{ND}\;=\;1\;-\;\frac{marker\;in\;diet\;\left(g/kgDM\right)\;\times \;N\;in\;faeces\;\left(g/kgDM\right)}{marker\;in\;faeces\;\left(g/kgDM\right)\;\times \;N\;in\;diet\;\left(g/kgDM\right)},$$

where N is the nutrient and ND the nutrient digestibility.

At the end of the trial, on the same day, animals were humanely slaughtered at a commercial slaughterhouse, where they were assessed by a trained veterinarian. Before being slaughtered, the scrotal neck and hind leg coronary band of each animal was assessed for normal appearance by a trained veterinarian, using a score based on the presence of localized swelling, ranging from 1 to 4 (1 = normal; 2 = slightly swollen; 3 = swollen and 4 = very swollen). Upon slaughter, pulmonary scores were assigned based on presence of parenchyma lesions, pulmonary consolidation in the cranial lobes, pleural adhesions and pleural fibrin deposition, as reported by Thompson [29]. The liver was examined to assess the presence of hepatic abscesses and hepatic scar tissue. As reported in Cozzi et al. [30], around thirty minutes after slaughter, the hot carcass weight (HCW) was recorded, as was the carcass conformation according to the European grading scheme defined SEUROP (S, E, U, R, O, P; from the best to the worst).

### 2.3. Meat Quality Analysis

From 30 carcasses, belonging to CON (*n* = 15) and LP (*n* = 15) diets, meat samples were collected and analyzed for meat quality. The 30 carcasses were selected by means of a simple randomization criterion from the two groups (CON and LP), after having discarded the outliers for ADG (animals with an ADG higher or lower than the average ± 3 standard deviations). From the 15 carcasses selected for each group, muscle samples were collected to assess the carcasses for meat quality. Briefly, boneless strip loin samples of m. *longissimus thoracis* (MLT) were excised with other adhering muscles, from the 4th to the 6th rib of the right side of carcasses (routine excision area to dissect the carcasses into quarters) 2 days post mortem, and samples were single vacuum packed and aged at 4 °C for other 5 days. Thus, after 7 days post mortem (2 days in carcass and 5 days in vacuum packed storage conditions) the joint samples of MLT were analyzed in order to determine the quality traits of fresh and cooked meat. A new cutting surface of a 2.0 cm thick of MLT was bloomed in the air at 3 ± 1 °C for approximately 60 min (1 h of blooming) and then instrumental color was recorded in plastic-wrapped meat samples according to the method suggested by the Commission Internationale de l’Éclairage (CIE) [31]. By the means of five replicates, the CIE-L*a*b* color coordinates (L*, lightness; a*, redness; and b*, yellowness) were measured using a Konica Minolta CM-600d Spectrophotometer (Konica Minolta INC, Osaka, Japan) with illuminant D65 and a 10° standard observer. The meat color was also detected after 24 h of exposition to air at 3 ± 1 °C. Weight loss during cooking was determined on 2.5 cm thick steaks excised from the MLT samples and wrapped individually in polyurethane bags. Samples were heated in a thermostatic (75 °C) water bath for 50 min and then cooled in running tap water for at least 40 min. Five cylindrical meat cores 1.25 cm in diameter were then excised from the cooked steak for the instrumental measurement of tenderness using a Warner-Bratzler shear force meter (Instron, High Wycombe, United Kingdom). Samples of meat from MLT were freeze-dried and ground to measure dry matter (DM) and intramuscular crude protein and fat content, the latter as ether extract [25].

### 2.4. Feeding Cost, Daily Gross Margin and Other Calculations

To calculate the possible economic benefits deriving from the use of low-protein diet, some of the determinants reported by Langemier [32] to compute cattle finishing profitability were considered. Specifically, the daily ration cost (DRC) for 1 kg of DM, the daily feed cost (DFC), the feed cost per kg of daily weight gain (CDG) and the daily gross margin (DGM, calculated on feeding cost) were calculated.

DRC, expressed in €/kg DM, was obtained according to the following equation:

$$\mathrm{DRC}\;=\;\sum_{i\;=\;1}^{n}\;\left(ci\;\times \;xi\right)$$

where i is the number of ingredients of the ration; c is the cost payed by the farmer for the ingredient i (€/kg DM); and x is the amount of the ingredient i in 1 kg DM of ration.

The daily feed cost, expressed as € spent daily for feeding one animal, was obtained according to the following equation:

DFC = DRC × DMI

where DRC is the cost of the ration, expressed as €/kg DM; and DMI is the average dry matter intake, expressed as kg of DM.

CDG, expressed in €/kg of daily weight gain, was obtained according to the following equation:

$$\mathrm{CDG}\;=\;\frac{DRC\;\times \;DMI}{ADG}$$

where DRC is the cost of the ration, expressed as €/kg DM; DMI is the average dry matter intake, expressed as kg of DM; and ADG is expressed in kg.

DGM, expressed in €, was obtained according to the following equation:

DGM = (V × ADG) − (DRC × DMI)

where V was the value (€) paid by the slaughterhouse for each kg of live weight, which in this case was 2.4 €/kg; ADG is expressed in kg; DRC, expressed in €/kg; and DM is the daily cost of the ration; DMI is expressed as kg of DM.

Furthermore, to estimate how efficiently the CP was used, crude protein intake (CPI), digestible crude protein intake (DCPI), crude protein fecal excretion, crude protein conversion ratio (CPCR) and digestible crude protein conversion ratio (DCPCR) values were calculated. To calculate CPI, DMI was multiplied by the CP% of the ration and CPCR was calculated dividing the average CPI by ADG, whereas DCPI and DCPCR were calculated multiplying CPD by CPI or CPCR, respectively. Crude protein fecal excretion was calculated by subtracting DCPI from CPI.

### 2.5. Statistical Analysis

Prior to statistical analysis, the categories of carcass conformation (SEUROP) were converted into numerical scores ranging from 1 (P) to 6 (S). Statistical analysis was computed using SAS software (2012, release 9.4; SAS Institute Inc., Cary, NC). For the whole dataset, the assumption of normality and variance homogeneity were tested using the Shapiro–Wilk test (PROC UNIVARIATE). Data on DMI, ADG, FCR, nutrient digestibility, CPI, DCPI, CPCR, DCPCR and feed costs were processed using PROC MIXED for repeated measures, with pen as a random effect and the autoregressive covariance structure. In the model, diet (CON versus LP), phase (P1 versus P2) and their interactions were used as fixed effects. The Bonferroni correction was used to make a post-hoc pairwise comparison. Data on lung, scrotal and coronary band scores were submitted to the non-parametric Mann–Whitney test to evaluate the effects of diets, whereas data on the liver score, expressed as percentages, were analyzed through a Z test. Carcass traits were submitted to a one-way ANOVA within PROC GLM to test the fixed effect of dietary CP level; in the case of meat quality data, the slaughter weight was included in the linear model as a covariate.

## 3. Results

At the beginning of the trial, the animals (345 ± 53 days old) weighed on average 486 and 484 kg for CON and LP, respectively (*p* = 0.755), but at the end of the trial, CON led to a significantly higher final weight (722 versus 705 kg, *p* = 0.044). As reported in Table 2, LP led to a 0.6 kg/d lower DMI (*p* < 0.05) and to a 0.11 kg/d lower ADG (*p* < 0.05), whereas diet had no significant effect on FCR. Passing from P1, the growing phase, to P2, the finishing phase, DMI (*p* < 0.01) and FCR (*p* < 0.001) increased on average of 1 kg and 1.95 points, respectively, whereas ADG showed a reduction of 0.23 kg/d (*p* < 0.001). No significant interaction between diet and phase was found.

As shown in Table 3, dietary protein level did not significantly affect the total tract digestibility of DM, CP, NDF or starch. On the other hand, the raising phase affected DMD (*p* = 0.001), CPD (*p* < 0.001), NDFD (*p* < 0.01) and starch_D (*p* < 0.05), which, except for starch_D, all significantly decreased in the finishing phase. No effect on digestibility was found for the interaction between diet and period.

As predicted, CPI (Table 4) was significantly lower in LP than in CON (*p* = 0.002), but no significant effect was found for CPCR. Taking into account the digestible protein ingested by animals, DCPI (*p* = 0.001) and DCPCR (*p* = 0.054) were both significantly lower in LP than in CON, whereas CP fecal excretion was not significantly affected. From a calculation of the daily feed cost of the two rations, it emerged that LP was 0.29 €/head/d less expensive than CON (*p* < 0.01), whereas CDG, the feed cost per kg of daily weight gain, and DGM, the daily gross margin calculated on feeding cost, were similar between the two diets. There was no significant interaction between diet and phase for any of the variables taken into account.

As a reflection of a higher final body weight, carcass weight was also significantly higher for CONs compared to LP diet bulls (*p* < 0.05), as reported in Table 5. Regarding the traits concerning the carcass, carcass weight that was higher for its conformation (SEUROP classification) was not affected by the dietary treatment (Table 5). Despite the effect of dietary CP level on DMI and ADG, the carcass classification was similar between treatments.

As reported in Table 6 and Table 7, the diet did not affect lung and coronary band scores or the occurrence of liver lesions, but did affect the scrotal score, which was significantly lower in LP than in CON.

Proximate composition and quality traits after heating treatment (cooking losses and tenderness) were not affected by the dietary CP nor by covariate carcass slaughter weight. Regarding meat color, L* and a* after one hour of blooming where both significantly lower for the LP diet compared with CON. However, no further differences were reported after 24 h (Table 8).

## 4. Discussion

Our main objective was to evaluate the impact of dietary protein concentration on production parameters, feed efficiency and meat quality of growing Charolais bulls. A secondary goal was to evaluate cost-effectiveness and farm profitability resulting from dietary protein reduction. The control diet used in this experiment was formulated as described by Cozzi et al. [30], in line with the typical feeding regime adopted by Italian specialized fatteners, both regarding the ingredients used and the protein and starch content of the ration. Diets were formulated to cover the high requirements of the growing and finishing phases of beef cattle breeds and were provided as total mixed rations (TMR) to diminish the risk of acidosis, using corn as the main crop included in the diet [30]. Italian, intensive Charolais cattle farms aim to attain an average daily gain of 1.5 kg/d [33,34] in 180–210 days, with a carcass yield of 60% [35].

As stated in different studies [36,37], an excess of protein beyond the requirements of the animal could lead to a waste of resources and may have negative repercussions also for the environment, since most of the protein in excess is excreted as NH_3_ in urine and feces [36,37]. The literature, in regard to the effect of the reduction of the protein level of diet on the performances of beef cattle in the finishing phase, is not very consistent, and sometimes, shows opposite results [37,38,39]. This is likely due to the fact that the optimal protein level for a finishing animal depends on many factors, among which there are breed, live weight, ADG, housing conditions, energy and NDF concentration of the ration and CP digestibility [40]. In a study conducted by Dal Maso et al. [17], reducing dietary CP concentration from 14.7% to 11.0% resulted in no variation in ADG, DMI or final weight in double-muscled Piemontese cattle. On the contrary, in Gleghorn et al. [38], feeding three different CP levels (11.5%, 13.0% and 14.5%) to British x Continental steers found the highest performance with 13% of CP. Furthermore, it was found that DMI differed only in the first 84 d of the trial. In addition, in a series of experiments conducted by Trenkle [41] in implanted continental crossbreed steers, animals fed 14% CP from the 90 d to the 112 d of the finishing period gained 9.9% more and had a higher DMI than implanted steers fed 11% CP. This is in line with our results, for which the highest ADG was attained with the high protein diet.

The reduction of DMI by LP bulls is likely the result of a reduced activity by ruminant microorganisms that have inadequate ruminal ammonia—N—available for fermentation and microbial synthesis [42]. As a consequence of a lower DMI, the LP diet also led to lower CPI and DCPI. In line with our findings, a study conducted on double-muscled Belgian Blue bulls, Valkeners et al. [43], reported that a reduction of the ruminal degradable protein balance from +5.3 to −23.7 g/kg DM led to a linear decrease of DMI and ADG; and Thomson et al. [44] found a linear increase in DM intake (DMI) and ADG with increasing CP levels in cattle fed high-concentrate diets. The decrease of the DMI was followed by the reduction of ADG which resulted in a lack of difference in feed efficiency (FCR).

Total tract apparent-digestibility of DM, NDF, CP and starch were not significantly affected by a lower level of CP, which agrees with what reported by De Amaral et al. [45] and Mariz et al. [42], who found that increasing the CP level from 10% to 14% has no effect on digestibility of nutrients in beef cattle diet. Another study [46] found that increasing CP level leads to higher NDF digestibility. This difference between the outcomes found in the literature can possibly be explained by the fact that the effect of CP levels on the digestibility of nutrients depends on many different factors, such as protein source, dietary CP level, animal condition and breed [47,48]. No significant differences were found in CP fecal excretion and this could be due to an imbalance between amino acid availability and requirements in the LP ration. The LP diet did not cause a significant variation of CPCR but did decrease the DCPCR, which refers to the amount of digested protein needed to gain 1 kg of BW. The reason why the DCPCR value for the LP diet tended to be significantly lower than the control diet would need further investigation to be explained, involving the analysis of fermentable metabolizable energy and effective rumen degradable protein, which were not measured in this trial. However, it is reasonable to think that this difference was likely due to an imbalance between degradable protein and available energy for microbes at the ruminal level [49]. Collectively, these results suggest that in Charolais bulls in their growing and finishing phases, CP requirements are not completely covered by 13.5% CP inclusion in the diet and that a more accurate balance between degradable energy and protein needs to be reached when feeding 15.0% of CP, to optimize DCPCR and decrease CP losses through fecal excretion.

In regard to the economic side of the experiment, it emerged that even though the LP diet was significantly cheaper than the CON diet of about 12.8%, it neither affected the daily feed cost per kg of growth, nor the daily gross margin, which is the difference between the farmer’s income for a kg of live BW and the feed cost. This result can be explained by the fact that LP bulls had a lower ADG than CON bulls. Although decreasing the CP level of Charolais bulls’ diet in the growing and finishing phases did not negatively affect the farm daily gross margin, it must be considered that, most commercial breeders want to send the animals to the slaughterhouse after having reached a determined target BW, and that to reach the final BW of CON bulls, LP bulls would need to be raised for 14 more days, delaying the possibility of raising new animals in the same facilities. As animals grow and reach their mature BW, there is a gradual change in their body composition that is characterized by a higher percentage of fat deposition, which increases the energy required to gain 1 kg of BW [38]. For this reason, in the finishing phase, DMI and FCR increase, whereas ADG decreases, compared with the growing phase.

Looking at the effect of the raising phase, lower DMD, CPD and NDFD values were reported during the finishing period, likely because as DMI increases and NDF concentration declines, the rate of passage of ingesta throughout the rumen increases, leading to a decline of total tract apparent digestibility. In fact, a higher transit rate of ingesta throughout the rumen lowers the time available for degradation and absorption of nutrients [50]. This explanation can also justify the higher CP fecal excretion found in the finishing phase. As a consequence of higher DMI and lower digestibility of nutrients and ADG, a higher FCR value was reported during the finishing phase, which can indeed explain the higher CDG registered in this period.

In regard to the significantly higher scrotal score recorded before slaughter in CON bulls, it is in line with the results reported by Brito et al. [51] and Kastelic et al. [52], who found that bulls in the finishing phase tend to accumulate fat in the scrotal neck, increasing their scrotal circumference when fed over-conditioning diets rich in starch and protein. In this study, only seven CON bulls had a scrotal score of 2, whereas all LP bulls had scrotal score 1, and there were no scores higher than 2. The fact that, even with the CON diet, bulls had on average a low scrotal score, was likely due to the relatively high level of NDF (29.4% of DM) used in this study; the high fat deposition in the scrotal neck is usually reached in bulls growing over 1.6 kg/d and fed very high energy diets (80% grain, 20% forage), as reported by Kastelic et al. [52] and Brito [53]. This diet was chosen, in fact, to attain good performance but avoid the onset of ruminal acidosis, that in ruminants is known to be enhanced by high starch and low fiber diets, due to a drop in rumen pH and a disequilibrium in the microbial population [54]. The fact that, in this study, the presence of liver abscesses was low (on average 6.90%), confirms that ruminal acidosis was properly prevented, since the incidence of liver abscesses in beef cattle farms generally ranges from 10% to 20% [55]. The other lesions detected at the slaughterhouse did not seem to relate to the protein level of the diet, and no significant differences were found between groups.

Concerning the carcass traits, except for the carcass weight, which was higher in the CON due to the higher ADG, the other parameters considered in this experiment were not affected by the dietary protein level. Although the reduction of protein percentage in the ration could have caused a lack of amino acids’ availability, the SEUROP classification was not affected. As stated by Fluharty et al. [56], carcass conformation is not affected by increased dietary protein level, if it is not associated with an increase in the nutritional level (metabolizable energy) of the diet. Indeed, morphological differences are related to the breed type and nutritive value of the diet, especially when both DMI and nutritional requirements are quite similar among dietary treatment [57]. Carcass classification is one of the main references in European beef market; thus, the outcomes of the study suggest the opportunity to reduce the dietary total protein percentage in a young bull rearing system without reducing this price-related carcass quality trait. Regarding the meat quality, no effects on muscle protein synthesis or on the tissue conformation, were recorded, inducing similar water holding capacities and tenderness. This lack of difference could be partially due to the very short ageing time (seven days post mortem). According to the literature [58] the intramuscular fat content was quite low because that is a specific trait of French, specialized beef breeds, such as Charolais. Contrary to the findings of Consolo et al., [59] and Madruga et al. [60] who also tested the effect of the dietary CP nested in different feeding regimens, in this study, the main effect in muscle quality traits was related to a change in meat color.

In relation to color, the LP diet promoted a significant decrease of lightness (L*) associated with an increase of redness (a*), probably due to a variation in the early redox dynamics of myoglobin, since there were no further differences in either L* or a* after 24 h of air exposition. This phenomenon could be related to a change of myoglobin structure and function that depends on the presence of specific amino acids (i.e., histidine), the incidence of its four major chemical forms and the interaction with the surface moisture and the intramuscular lipids [61].

## 5. Conclusions

Based on the results, it can be concluded that lowering the protein level from 15% to 13.5% of DM, during the growing and finishing phases in young Charolais bulls, raised according to the Italian system, does not appear to significantly change the ration digestibility, the crude protein fecal excretion, the feeding cost per kg of growth or the daily gross margin, but it reduces ADG, and therefore lengthens the raising period required to reach a determined final body weight. The lower inclusion of dietary CP did not affect carcass and meat quality traits, except for a moderate change in the meat color due to a relatively early higher redness at blooming. By this study, it is possible to conclude that the CP requirements to maximize the performance of Charolais bulls during growing and finishing phases are higher than 13.5% of dry matter, even though this low crude protein level does not affect the daily gross margin and the overall meat quality traits. More studies are needed to deepen the knowledge on the balance between protein and energy requirements of Charolais bulls during the growing and finishing phases, focusing possibly on metabolizable protein requirements.

## Figures and Tables

**Table 1:** Ingredients, proximate composition and daily cost of the ration (DRC) of diets (low protein, LP; control, CON) fed to the young Charolais bulls during backgrounding, transition, growing (P1) and finishing (P2) phases at Rovigo Province, Italy.

Ingredients and Composition	Backgrounding (BKG) and Transition (TST) Diet		Growing and Finishing Diets			
	BKG	TST	CON		LP	
			P1	P2	P1	P2
Ingredients (% of DM)						
Maize silage	18.9	12.2	22.4	22.2	23.6	23.4
Pressed beet pulps	10.9	9.98	17.2	17.8	17.0	17.8
Straw	-	-	6.30	4.90	6.60	5.10
Meadow hay	14.1	9.08				
Maize meal	-	14.5	20.1	25.1	23.4	27.4
Weat bran	25.8	21.3	15.7	12.7	15.5	13.2
Soybean meal	20.1	23.9	10.5	10.0	5.80	5.50
Vitamin and mineral mix	10.2	9.04	7.80	7.30	8.05	7.55
DRC (€/kg DM)			0.212	0.213	0.197	0.198
Proximate composition						
DM (%)	61.1	56.7	49.7	53.7	48.4	52.8
CP (% of DM)	14.1	14.0	14.9	14.7	13.6	13.4
EE (% of DM)	2.33	2.63	3.32	3.13	3.33	3.15
NDF (% of DM)	38.2	35.2	32.7	29.0	33.0	29.2
Starch (% of DM)	26.1	29.8	29.1	33.5	30.4	34.8
ME (Mj/kg DM)	10.8	10.0	10.8	11.1	10.8	11.1

DM: dry matter; CP: crude protein; EE: ether extract; NDF: neutral detergent fiber; ME: metabolizable energy.

**Table 2:** The effects of high protein (CON) and low protein (LP) diets, on dry matter intake (DMI), average daily gain (ADG) and feed conversion ratio (FCR) by young Charolais beef bulls at Rovigo Province, Italy, during growing (P1) and finishing (P2) phases. Data are the means of 5 or 6 pens and 9 to 10 animals per pen.

Items	CON	LP	P1	P2	CON		LP		SEM	*p*-Value		
					P1	P2	P1	P2		Diet	Phase	Diet × Phase
DMI (kg/d)	10.6	10.0	9.8	10.8	10.1	11.1	9.5	10.4	0.55	0.036	<0.05	0.816
ADG (kg/d)	1.47	1.36	1.53	1.30	1.59	1.36	1.48	1.24	0.08	0.016	<0.001	0.773
FCR	7.29	7.50	6.42	8.37	6.38	8.19	6.46	8.54	0.64	0.523	<0.001	0.680

**Table 3:** Effect of high protein (CON) and low protein (LP) diets, during growing (P1) and finishing (P2) phases, on the digestibility of dry matter, crude protein, neutral detergent fiber and starch (DMD, CPD, NDFD and starch_D, respectively) by young Charolais beef bulls at Rovigo Province, Italy. Data are the means of 5 or 6 pens and 9 to 10 animals per pen.

Items	CON	LP	P1	P2	CON		LP		SEM	*p*-Value		
					P1	P2	P1	P2		Diet	Phase	Diet × Phase
DMD	62.0	61.8	64.8	59.0	64.9	59.2	64.8	58.8	1.28	0.861	0.001	0.917
CPD	52.8	50.6	58.8	44.6	60.3	45.2	57.3	43.9	2.26	0.410	<0.001	0.655
NDFD	42.1	43.4	45.5	40.1	44.3	40.0	46.7	40.2	1.56	0.406	0.006	0.484
Starch_D	96.4	96.5	95.5	97.4	95.3	97.5	95.8	97.2	0.70	0.878	0.022	0.579

**Table 4:** The effects of high protein (CON) and low protein (LP) diets during growing (P1) and finishing (P2) phases, on crude protein (CP) intake (CPI), digestible CPI (DCPI), CP conversion ratio (CPCR), digestible CPCR (DCPCR), daily feed cost (DFC), cost of daily gain (CDG) and daily gross margin (DGM) values of young Charolais beef bulls at Rovigo Province, Italy.

Items	CON	LP	P1	P2	CON		LP		SEM	*p*-Value		
					P1	P2	P1	P2		Diet	Phase	Diet × Phase
CPI (kg/d)	1.57	1.35	1.40	1.52	1.50	1.63	1.30	1.40	0.039	0.002	<0.001	0.451
CP faecal excretion (g/d)	0.746	0.671	0.575	0.842	0.596	0.895	0.553	0.788	0.040	0.131	<0.001	0.297
DCPI (kg/d)	0.825	0.679	0.827	0.676	0.909	0.740	0.745	0.611	0.034	0.001	<0.001	0.590
CPCR	1.08	1.01	0.915	1.17	0.951	1.20	0.878	1.14	0.046	0.208	<0.001	0.876
DCPCR	0.560	0.499	0.539	0.520	0.575	0.544	0.503	0.496	0.023	0.054	0.498	0.660
DFC (€)	2.26	1.97	2.01	2.22	2.14	2.37	1.88	2.07	0.057	0.004	<0.001	0.468
CDG (€/kg growth)	1.55	1.48	1.31	1.72	1.35	1.74	1.27	1.69	0.067	0.370	<0.001	0.820
DGM (€)	1.28	1.29	1.67	0.900	1.66	0.900	1.68	1.90	0.118	0.943	<0.001	0.937

**Table 5:** The effects of high protein (CON) and low protein (LP) diets on carcass weight, carcass yield and SEUROP-carcass classification.

Carcass Traits	CON	LP	*p*-Value
Carcass weight (kg)	437	425	0.026
Carcass yield (%)	60.7	60.3	0.488
SEUROP	4.26	4.29	0.827

SEUROP: S = 6, E = 5, U = 4, R = 3, O = 2, P = 1.

**Table 6:** The effects of high protein (CON) and low protein (LP) diets on lung, scrotal and coronary band scores.

Items	CON	LP	*p*-Value
Lung score	1.96 (2.66)	2.22 (1.99)	0.314
Scrotal score	1.12 (0.329)	1.00 (0.00)	0.025
Coronary band score	1.21 (0.409)	1.23 (0.425)	0.670

Data are expressed as average and (standard deviation).

**Table 7:** The effects of high protein (CON) and low protein (LP) diets on liver lesions.

Liver Lesions	CON	LP	*p*-Value
Lymphadenopathy (%)	3.33	7.14	0.990
Liver abscesses (%)	3.33	10.7	0.225
Liver scars (%)	36.7	42.8	0.990

**Table 8:** The effects of high (CON) and low (LP) protein and covariate slaughter weights on the meat quality of young Charolais beef bulls at Rovigo Province, Italy.

Meat Quality Traits	Unit	Diet		SEM	*p*-Value	
		CON	LP		Diet	Slaughter Weight
Physical traits						
Cooking loss	%	26.7	27.4	0.01	0.337	0.144
Maximum shear force	N	33.2	33.3	1.44	0.941	0.239
Meat composition (wet weight)						
Moisture	g/100 g	75.7	75.9	0.30	0.591	0.622
Crude protein	g/100 g	19.7	19.9	0.24	0.674	0.615
Ether extract	g/100 g	2.90	2.70	0.25	0.688	0.456
Color (after 1 h of blooming)						
Lightness, L*		44.9	43.1	0.40	0.004	0.665
Redness, a*		13.3	14.2	0.28	0.009	0.274
Yellowness, b*		11.7	11.8	0.25	0.637	0.427
Color (after 24 h)						
Lightness, L*		44.8	44.3	0.45	0.433	0.483
Redness, a*		16.1	15.2	0.43	0.171	0.932
Yellowness, b*		13.2	12.7	0.29	0.233	0.719
